# Could Serum TSH Levels Predict Malignancy in Euthyroid Patients Affected by Thyroid Nodules with Indeterminate Cytology?

**DOI:** 10.1155/2020/7543930

**Published:** 2020-04-21

**Authors:** Carlo Cappelli, Ilenia Pirola, Elena Gandossi, Mario Rotondi, Davide Lombardi, Claudio Casella, Fiorella Marini, Maura Saullo, Barbara Agosti, Elena Di Lodovico, Luca Chiovato, Alberto Ferlin, Maurizio Castellano

**Affiliations:** ^1^Department of Clinical and Experimental Sciences, SSD Medicina ad Indirizzo Endocrino-metabolico, University of Brescia, ASST Spedali Civili di Brescia, 25123 Brescia, Italy; ^2^Unit of Internal Medicine and Endocrinology, ICS Maugeri IRCCS, Laboratory for Endocrine Disruptors, University of Pavia, Pavia, Italy; ^3^Department of Otorhinolaryngology—Head and Neck Surgery, University of Brescia, Brescia, Italy; ^4^Department of Molecular and Translational Medicine, University of Brescia, Brescia, Italy

## Abstract

**Background:**

Serum TSH levels in the upper-normal range were reported to be associated with increased risk of thyroid malignancy. However, measurement of TSH levels is currently not recommended for assessing the risk of malignancy in patients with newly diagnosed thyroid nodules.

**Objective:**

To evaluate a possible relationship between the serum levels of TSH and the histological outcome of patients undergoing thyroidectomy for thyroid nodules with indeterminate cytology.

**Materials and Methods:**

We collected the clinical data of all patients who had performed ultrasound-guided FNA of thyroid nodules with cytological diagnosis of indeterminate lesions (TIR3A and TIR3B) and serum TSH levels within the normal range. All patients had been submitted to thyroid surgery (hemi or thyroidectomy, as appropriate), and histological diagnosis had been performed.

**Results:**

A histological diagnosis of thyroid malignancy was rendered in 74/378 (19.6%) nodules. Patients with histologically proven thyroid malignancy were characterized by higher serum levels of TSH as compared to patients with histologically proven benign nodules (3.03 ± 1.16 vs. 2.37 ± 1.19 mIU/L, *p* < 0.001). To further analyze the role of serum TSH in predicting thyroid cancer, patients were stratified in 4 groups according to quartiles of TSH concentrations. The prevalence of malignancy was 12.2% for the first quartile and 50.0% for the last quartile. ROC curve analysis identified that a serum TSH level of ≥2.7 mIU/L predicted thyroid malignancy with a sensitivity of 61% and a specificity of 65%.

**Conclusions:**

TSH levels in the upper-normal range are associated with an increased risk of thyroid malignancy in patients affected by thyroid nodules with indeterminate cytology at FNA. The measurement of serum TSH levels represents an easily performed additional tool for decision-making in patients with indeterminate cytological findings.

## 1. Introduction

Recent surveys adopting ultrasound images showed thyroid nodule(s) in up to 70% of randomly selected subjects with higher frequencies in women and elderly patients, making it the most frequent endocrine disease today [1, 2]. The two objectives in thyroid nodule management are as follows: first, to evaluate if its presence is associated with or is the cause of thyroid function alteration; second, to exclude malignancy [3]. International guidelines clearly indicate which nodules should undergo fine needle aspiration cytology (FNA) [4–6]. FNA is clinically safe, cost-effective, minimally invasive, and has few complications [7]. Its limitations arise for nodules that are reported as showing indeterminate cytology (TIR3) because, even if at a relatively low rate, malignancy cannot be excluded. In such cases, molecular testing can represent an opportunity to identify thyroid cancer, although it cannot guarantee a correct diagnosis, and it also has relatively high costs [8].

Recently, the usefulness of serum thyreotropin (TSH) levels has been evaluated as a predictor of malignancy in thyroid nodules, demonstrating that higher serum TSH levels are associated with an increased risk of thyroid cancer [9]. This simple and free of adjunctive costs test for stratifying the risk of malignancy associated with a thyroid nodule was not previously evaluated in thyroid nodules with indeterminate cytology.

For this reason, the aim of this retrospective study was to evaluate the role of serum TSH in predicting malignancy in thyroid nodules with indeterminate cytology.

## 2. Materials and Methods

We reviewed the medical records of patients who had ultrasound-guided FNA of thyroid nodules at our department between September 2014 and February 2018. Only patients with indeterminate cytology and serum TSH values within the normal range of our laboratory, obtained at least one month before FNA, were enrolled. In addition, we selected only patients who had been submitted to hemi or thyroidectomy and followed in our institute, as appropriate. Patients in levothyroxine substitutive therapy and those in metformin treatment, in view of the TSH-lowering effect of metformin, were excluded from the study [10, 11]. The cytologic diagnoses were made in accordance with the last Italian consensus for the classification and reporting of thyroid cytology [12]. Indeterminate cytology (TIR3) was distinguished, on the basis of architectural and cytological alterations, into two sub-classes at different risks of malignancy: TIR3A (low-risk indeterminate lesion, LRIL) and TIR3B (high-risk indeterminate lesion (HRIL)) [12].

The final histological diagnosis on surgical specimens (according to World Health Organization Guidelines) was considered to be the goal standard, and it was carried out for all selected samples.

Serum concentrations of TSH (normal range: 0.4 ± 4.5 mIU/L and analytical sensitivity: 0.004 mIU/L) were measured using a fully automated Architect i2000 analyzer (Abbott Diagnostics, Abbott Park, IL, USA) using chemiluminescent magnetic immunoassays.

All patients provided written informed consent as to their enrolment in this study and for the storage and use of their data. The study was approved by the Local Ethical Committee (no. 3412).

### 2.1. Statistical Analysis

All data were collected in an electronic case report database. Comparisons between groups and difference between proportions were calculated using χ2 for categorical variables and ANOVA test for quantitative variables, as appropriate. A binary logistic regression analysis was performed to examine the influence of confounders on TSH levels between patients with or without malignancy. A ROC analysis was performed for TSH levels and the presence of malignancy. Two-tailed *p* < 0.05 was considered statistically significant. Statistical analyses were performed using SPSS 20.0 software (SPSS, Inc., Evanston, IL, USA).

## 3. Results

Between September 2014 and February 2018, 4321 nodules were submitted to fine needle aspiration cytology in our department. Among them, 350 (8.1%) lesions were nondiagnostic, 298 (6.9%) malignant, 3181 (73.6%) benign, and 492 (11.4%) displayed an indeterminate cytology (TIR3). We identified, in accordance with enrolling roles, 378 nodules from 378 patients that had been submitted to surgical treatment and followed in our institute with normal serum TSH levels obtained no more than one month before FNA.  Table 1 shows clinical and pathological characteristics of the 378 patients. The majority of patients (mean age of 54.7 ± 13.9 years old) were female 271/378 (71.7%). All the subjects were in euthyroidism (TSH 2.50 ± 1.21 mIU/L) in accordance with the enrolment rules, with a mean nodular size of 19.3 ± 9.2 mm.

Specifically, no difference emerged between TIR 3A and TIR 3B categories for gender (126/46 vs 145/61 F/M, *p*=0.308), age (53.7 ± 12.9 vs 55.1 ± 11.9 yrs, *p*=0.274), nodular size (mm) (19.2 ± 10.1 vs 19.4 ± 9.9, *p*=0.846), and serum TSH levels (2.53 ± 1.31 vs 2.47 ± 1.13 mIU/L, *p*=0.620), respectively.

Histologic evaluation revealed malignancy in 74/378 (19.6%) nodules, whereas 49 (13%) patients showed features accompanying chronic lymphocytic thyroiditis. The presence of lymphocytic thyroiditis was superimposable between TIR3A and TIR3B categories 20/172 (11.6%) vs 29/206 (14.1%, *p*=0.291) and not associated to thyroid cancer (38/304 (12.5%), benign vs 11/74 (14.9%) malignant lesions, *p*=0.354). In addition, no difference in TSH values was observed among patients with (49 subjects) or without (329 patients) chronic lymphocytic thyroiditis (2.2 ± 1.29 mIU/L vs 2.5 ± 1.20 mIU/L, respectively, *p*=0.079).

**Figure 1:**
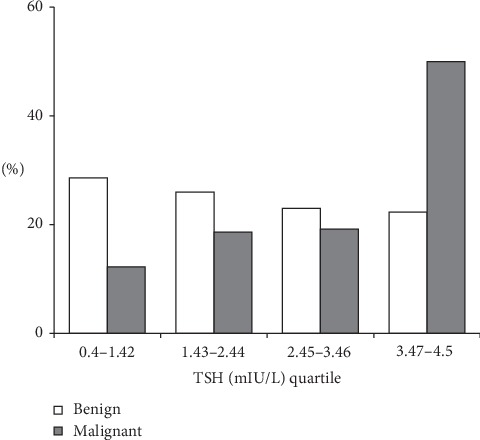
Frequencies of malignant and benign nodules according to TSH quartile.

**Figure 2:**
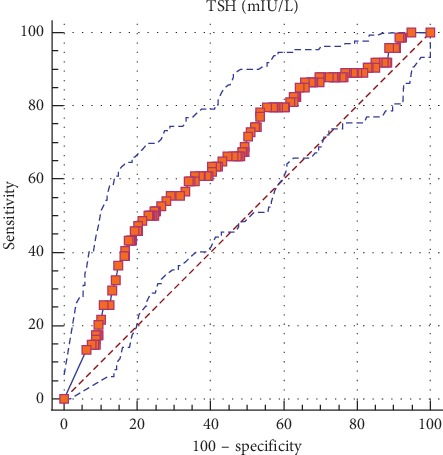
Roc curve analysis of TSH levels for the detection of thyroid cancer.

The rate of cancer was significantly lower in TIR3A than TIR3B lesions (17/172 (9.8%) vs. 57/206 (27.4%), *p* < 0.0001, respectively). Patients with malignancy evidenced higher serum TSH levels than those with histological proven benign nodules (3.03 ± 1.16 vs. 2.37 ± 1.19 mIU/L, *p* < 0.001). The difference remained significant after adjusting for possible confounders (Table 2). To better analyze the role of serum TSH as a predictor of thyroid cancer, we subdivided the sample into 4 quartiles of similar size according to patients' TSH values (quartile 1: 0.4 ≥ TSH ≤ 1.42 mIU/L; quartile 2: 1.42 > TSH ≤ 2.44 mIU/L; quartile 3: 2.44 > TSH ≤ 3.46 mIU/L; and quartile 4: 3.46 > TSH ≤ 4.5 mIU/L). The prevalence of malignancy was 12.2% for the first quartile and 50.0% for the last quartile (Figure 1). This high rate of malignancy in the last quartile was evidenced both in TIR3A (7/17) than in TIR3B (27/57) nodules. The ROC curve analysis indicated that a TSH value of ≥2.7 mIU/L identified patients with malignancy with a sensitivity of 61% and a specificity of 60% (Figure 2).

**Figure 3:**
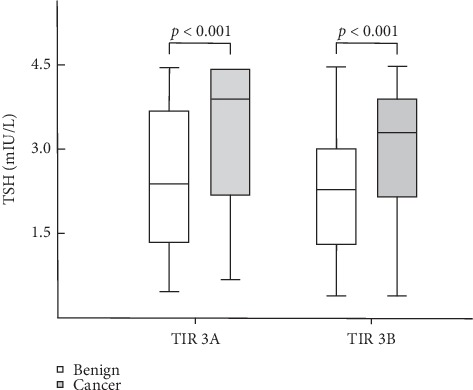
TSH level in malignant and benign thyroid lesions among patients with TIR3A and TIR3B nodules.

Subsequently, we analyzed the TSH values separately in patients with nodules TIR3A and TIR3B (Figure 3). Specifically, subjects with thyroid cancer showed higher serum TSH levels than those with benign lesions, both in subjects with cytological TIR3A nodules (2.45 ± 1.29 vs. 3.29 ± 1.26 mIU/L, *p* < 0.001) and in those with TIR3B (2.28 ± 1.08 vs. 2.95 ± 1.13 mIU/L, *p* < 0.001). The prevalence of malignancy was 5.9%, 23.3%, 12%, and 58.8% in the different quartiles for TIR3A and 12%, 19.5%, 21.1%, and 47.4% for TIR 3b, respectively. The ROC curve showed that a TSH ≥3.8 mIU/L detected cancer in TIR3A patients with a sensitivity and a specificity of 59% and 79%; whereas, a TSH ≥2.68 mIU/L in TIR3B subjects identified malignancy with a sensitivity of 65% and a specificity of 63%.

## 4. Discussion

The present retrospective study showed a relationship between serum TSH levels and the risk of malignancy in patients affected by nodules with indeterminate cytology.

FNA is currently the most simple, accurate, safe, and cost-effective method of identifying malignant thyroid nodules [4, 7, 13]. Unfortunately, 15% to 30% of aspirations yield in the “gray zone” of indeterminate findings in which malignancy cannot be excluded [14]. For this reason, many patients are submitted to thyroid surgery even if the majority prove to have a histological benign disease [4, 14, 15]. Many strategies have been reported to shed a light onto this “gray zone”. The British Thyroid Classification and Bethesda Reporting System for Thyroid Cytology divide the indeterminate category into two sub-groups with different risks of malignancy reducing, at the same time, the rate of diagnostic surgery [16, 17]. Recently, an Italian consensus has further stratified TIR3 lesions into two categories, TIR3A and TIR3B, on the basis of architectural and cytological alterations. In many patients with TIR3A, a clinical follow-up, mainly based upon ultrasound surveillance, is the preferred option due to the low risk of cancer, even if malignancy cannot to be excluded [12].

In this case, molecular testing to detect specific gene mutations and gene rearrangements aimed at identifying malignancy can represent an opportunity. Although molecular testing are showing great promise in reducing the diagnostic uncertainty of cytologically indeterminate thyroid nodules, reducing the surgical rates for these lesions remain too expensive on the routine clinical management for a part of laboratories till today [8, 18–22].

The role of serum thyreotropin levels in the development and/or progression of thyroid cancer remains controversial. Indeed, it is well known that TSH is a major thyroid cell growth factor and that thyreotropin-activated signaling pathways play a role in thyroid tumorigenesis [9, 23]. In addition, TSH suppression is a crucial therapeutic tool in, at least some, patients affected by differentiated thyroid cancer [4]. On the other hand, several studies have evaluated the usefulness of serum TSH levels as a predictor of malignancy in thyroid nodules in the last years with conflicting results [9, 24–28]. Recently, Golbert et al. have shown, in a large series of patients, that higher thyreotropin levels are associated with an increased risk of thyroid cancer [9]. This was confirmed in a prospective cross-sectional study by Duccini and colleagues. The authors showed that higher TSH levels within the reference range were positively associated with the diagnosis of differentiated thyroid cancer in thyroid nodules [29]. This is in line with previous evidence in euthyroid patients [30–32]. However, although the 2016 ATA guidelines acknowledge that upper normal serum TSH levels are associated with an increased risk of malignancy, as well as more advanced stage thyroid cancer, measurement of TSH levels has not yet been recommended as a tool for stratifying the risk of malignancy in patients with newly diagnosed thyroid nodules [4].

To the best of our knowledge, no previous studies have investigated the role of TSH within the normal range in predicting malignancy of patients with thyroid nodules with indeterminate cytology. Our study, summarized in a poster presentation at the 42nd Annual Meeting of the European Thyroid Association [33] showed, for the first time, that those subjects with malignant nodules presented higher serum TSH levels than patients with benign lesions. Accordingly, the prevalence of malignancy was higher in subjects with TSH levels ≥2.7 mIU/L, as defined via ROC curve analyses.

In particular, indeterminate cytological lesions with a low risk of malignancy (i.e. TIR3A) represent the deeper “gray zone.” As a matter of fact, just as TIR3B should be submitted to surgery, TIR3A likely should not. Having a simple, affordable test within the reach of all physicians, to help to stratify the risk of malignancy, in particular for TIR3A nodules, could be of great interest. In these patients, our data suggested that with increasing levels of thyreotropin, a significant increase in the risk of malignancy occurs. Accordingly, ROC curve analyses showed that the prevalence of malignancy was higher in subjects with TSH values ≥3.8 mIU/L.

The present study has some limitations. First, the absence of information about thyroid antibodies and the use of a single TSH measurement. Notably, 49 patients showed features accompanying chronic lymphocytic thyroiditis. This characteristic resulted was not associated to thyroid cancer. Moreover, all patients had serum TSH levels within the normal ranges of our laboratory, obtained at least one month before FNA. However, the sample size of our study and, moreover, the prevalence of malignancy observed both in TIR3A and TIR3B nodules superimposable with recent literature data [34, 35] strengthen our results.

## 5. Conclusion

Higher TSH levels are associated with an increasing risk of malignancy in patients affected by thyroid nodules with indeterminate cytology. The use of TSH can represent an easy adjunctive diagnostic test for decision-making in patients with indeterminate cytological findings.

## Figures and Tables

**Table 1:** Clinical and pathological characteristics of the patients.

	TIR3 (*n* = 378) *n*. (%)	TIR3A (*n* = 172) *n*. (%)	TIR3B (*n* = 206) *n*. (%)	*p* value
Gender				
Females	271 (71.7%)	126 (73.2%)	145 (70.4%)	0.308
Males	107 (28.3%)	46 (26.8%)	61 (29.6%)	
Age (yrs)	54.7 ± 13.9	53.7 ± 12.9	55.1 ± 11.9	0.274
BMI (kg/m^2^)	27.4 ± 3.3	27.2 ± 3.1	27.1 ± 3.6	0.775
Nodular size (mm)	19.3 ± 9.2	19.2 ± 10.1	19.4 ± 9.9	0.846
TSH (mIU/L)	2.50 ± 1.21	2.53 ± 1.31	2.47 ± 1.13	0.620

**Table 2:** Logistic regression analysis of thyroid malignancy in the study population.

Variable	Odds ratio (95% CI)	*p* value
TSH	1.61 (1.28–2.03)	<0.001
BMI	0.99 (0.87–1.12)	0.908
Age	1.01 (0.98–1.02)	0.689
Hashimoto thyroiditis	1.37 (0.64–2.90)	0.408

CI, confidence interval.

## Data Availability

The clinical data used to support the findings of this study are available from the corresponding author upon request.
